# Assessment of the Antimicrobial Activity of Algae Extracts on Bacteria Responsible of External Otitis

**DOI:** 10.3390/md13106440

**Published:** 2015-10-20

**Authors:** Gianluca Pane, Gabriele Cacciola, Elisabetta Giacco, Gian Luigi Mariottini, Erika Coppo

**Affiliations:** 1 Dipartimento di Scienze Chirurgiche e Diagnostiche Integrate (DISC), Sezione di Otorinolaringoiatria, Università di Genova, Largo R. Benzi 10, Genova 16132, Italy; E-Mail: Gianluca.Pane@unige.it; 2 Dipartimento di Scienze Chirurgiche e Diagnostiche Integrate (DISC), Sezione di Microbiologia, Università di Genova, Largo R. Benzi 10, Genova 16132, Italy; E-Mails: gabrielecacciola@live.it (G.C.); erika.coppo@unige.it (E.C.); 3 Dipartimento di Scienze della Terra, dell’Ambiente e della Vita (DISTAV), Università di Genova, Viale Benedetto XV 5, Genova 16132, Italy; E-Mail: Elisabetta.Giacco@unige.it

**Keywords:** external otitis, biofilm, algae, antimicrobial susceptibility, antibiotic activity, *Pseudomonas aeruginosa*, *Staphylococcus aureus*, *Dunaliella tertiolecta*, *Pseudokirchneriella subcapitata*

## Abstract

External otitis is a diffuse inflammation around the external auditory canal and auricle, which is often occurred by microbial infection. This disease is generally treated using antibiotics, but the frequent occurrence of antibiotic resistance requires the development of new antibiotic agents. In this context, unexplored bioactive natural candidates could be a chance for the production of targeted drugs provided with antimicrobial activity. In this paper, microbial pathogens were isolated from patients with external otitis using ear swabs for over one year, and the antimicrobial activity of the two methanol extracts from selected marine (*Dunaliella salina*) and freshwater (*Pseudokirchneriella subcapitata*) microalgae was tested on the isolated pathogens. Totally, 114 bacterial and 11 fungal strains were isolated, of which *Staphylococcus* spp. (28.8%) and *Pseudomonas aeruginosa* (*P. aeruginosa*) (24.8%) were the major pathogens. Only three *Staphylococcus aureus* (*S. aureus*) strains and 11 coagulase-negative Staphylococci showed resistance to methicillin. The two algal extracts showed interesting antimicrobial properties, which mostly inhibited the growth of isolated *S. aureus*, *P. aeruginosa*, *Escherichia coli*, and *Klebsiella* spp. with MICs range of 1.4 × 10^9^ to 2.2 × 10^10^ cells/mL. These results suggest that the two algae have potential as resources for the development of antimicrobial agents.

## 1. Introduction

External otitis (EO) is the inflammation or infection of the external auditory canal (EAC), which may also involve the tympanic membrane [1]. Usually, EO is due to bacterial infection, but less frequently it is caused by fungi or it is associated to local or systemic non-infectious processes [2,3,4]. *Staphylococus aureus* (*S. aureus*) and *Pseudomonas aeruginosa* (*P. aeruginosa*) are the main etiological agents of the disease, but other Gram-negative bacteria, such as *Escherichia coli* (*E. coli*) and *Klebsiella* spp., can be involved [1,5]. The exposition of EAC to water, humidity, local traumatism, occlusion, or the occurrence of dermatitis can increase the susceptibility to infections.

The annual incidence of EO in humans is included between 1:100 and 1:250, with regional variations depending on age and geographical location [1].

Otorrhea and otalgia are characteristic symptoms of EO. Otalgia can vary from simple pruritus to severe pain exacerbated by ear movements, for example during mastication [2]. Congestion, hyperemia and edema of EAC are often associated to suppurating otorrhea. In the case of swelling with obstruction of EAC induced by inflammation, or when otorrhea is abundant, patients can also suffer from hearing loss [2]. The diagnosis of acute EO is done clinically, by otoscopy, otomicroscopy, tympanometry, and by microbiological analysis of ear swabs for the identification of pathogens [6,7].

The therapy of bacterial EO is based on the administration of antibiotics. Penicillinase-resistant penicillins, cephalosporins and fluoroquinolones are suggested for oral or parenteral therapy, while quinolones have mainly topical utilization [2,8]. Antibiotics can also be associated with anti-inflammatory steroids and/or analgesics [7]. Notably, the administration of oral antibiotics is indicated for cases in which the infection has spread beyond the EAC or when the risk of a rapid progression of the infection occurs [2,9].

The utilization of natural bioactive compounds in pharmacology is known to be an efficacious procedure. As a matter of fact, extracts from organisms (plants and animals) and microorganisms (bacteria, algae, fungi) are well known sources of compounds provided with interesting biological and therapeutic properties [10,11]. For example, more than 75% of drugs utilized to treat infectious diseases are derived from natural sources [12].

From this point of view, algae have been demonstrated to produce secondary metabolites other than those produced by terrestrial organisms [13]. Therefore, they have been indicated to be a source of compounds of biomedical interest [14,15,16].

*Dunaliella tertiolecta* (*D. tertilolecta*) (*Chlorophyta*: *Chlorophyceae*) is a unicellular flagellate marine green alga. The cell is ellissoidal, oval or pear-shaped, and colorless [17]. *Pseudokirchneriella subcapitata* (*P. subcapitata*), formerly *Selenastrum capricornutum* (*Chlorophyta*: *Chlorophyceae*), is a motionless freshwater half moon-shaped green alga, which is easily culturable *in vitro*. It is widely employed as test-organism in ecotoxicology [18,19,20].

In previous studies, algae belonging to the genus *Dunaliella* (*Dunaliella salina*) were shown to produce antibiotic substances, mainly active on *E. coli*, *P. aeruginosa*, *S. aureus*, *Candida albicans*, and *Aspergillus niger* (*A. niger*) [21,22], and the butanol extract was shown to be active on *P. aeruginosa* and *Klebsiella pneumoniae* [23]. Furthermore, the occurrence of several antibiotic substances inhibiting the growth of *S. aureus* was observed in *Dunaliella primolecta* [24]. In another study, this algal extract has been recorded to have a similar antibacterial activity against MRSA [25]. For antimicrobial properties of *D. tertiolecta* extracts, a limited number of data exist and an activity against *E. coli*, *P. aeruginosa*, and *S. aureus* was not observed [26]. As far as we know, data about antimicrobial properties of *P. subcapitata* extracts have not been reported.

The aim of this research was the identification of the etiological agents of EO in ear swabs, the assessment of biofilm production, the susceptibility/resistance to antibiotics, and the evaluation of the antimicrobial activity of the two algal extracts towards the most common pathogens that cause EAC infections. During the study, which was conducted from January 2012 to January 2013, the microbiological evaluation and the characterization of the microbial charge in samples collected from patients with suspected EO was carried out. Taking into account the antimicrobial potential of extracts from natural sources, the experimental goal of this study was to assess the activity of extracts obtained from cultures of marine (*Dunaliella tertiolecta*) and freshwater (*Pseudokirchneriella subcapitata*) algae, with the purpose of improving the knowledge about the usefulness of these widely available, easy to obtain, and cost-effective natural extracts against human pathogens.

## 2. Results

### 2.1. Bacterial Isolates

A total of 100 ear swabs were collected from patients (58% males, 42% females) with suspected EO. The age of patients ranged from 15 to 92 years (mean age 48.1 years); 84 ear swabs were positive for microorganisms, whereas 16 were negative.

A total of 125 microorganisms were found, including 65 Gram-negative bacteria, 49 Gram-positive bacteria and 11 fungi. Table 1 summarizes the list and distribution of pathogens isolated from the positive ear swabs.

*P. aeruginosa* had the highest number of isolates (31), followed by *S. aureus* (14), *S. epidermidis* (14), and *E. coli* (8). Among Gram-negative bacteria, 33.8% were *Enterobacteriaceae*, 63.1% non-*Enterobacteriaceae*, and 3.1% *Vibrionaceae*. The majority of Gram-positive strains were Staphylococci (75.5%). Among Fungi, 54.5% were *Candida* spp., while the remaining 45.5% were *A. niger*.

**Table 1:** Microorganisms isolated from positive ear swabs. The number of isolates are reported in brackets. * = *Staphylococcus epidermidis* (14), *Staphylococcus capitis* (4), *Staphylococcus xylosus* (2), *Staphylococcus haemolyticus* (1), *Staphylococcus symulans* (1); ^^^ = *K. pneumoniae* (5), *Klebsiella oxytoca* (2); ^§^ = *Proteus mirabilis* (2), *Enterobacter cloacae* (2), *Enterobacter aerogenes* (1), *Rautella terrigena* (1), *Serratia marcescens* (1); ^#^ = *Ralstonia paucula* (2), *Achromobacter xylosoxydans* (2), *Acinetobacter baumannii* (1), *Pseudomonas alcaligenes* (1), *Burkordelia cepacia* (1), *Ralstonia picketti* (1), *Sphingomonas paucimobilis* (1), *Aeromonas hydrophyla* (1).

	Strain	Isolated Strains (No.)	%
**Gram-Positive Bacteria**	*Staphylococcus aureus*	14	11.2
	CoNS *	22	17.6
	*Enterococcus* spp.	1	0.8
	*Kokuria* spp.	2	1.60
	*Streptococcus pneumoniae*	2	1.60
	*Micrococcus* spp.	8	6.40
**Gram-Negative Bacteria**	*Pseudomonas aeruginosa*	31	24.8
	*Escherichia coli*	8	6.4
	*Klebsiella* spp. ^^^	7	5.6
	Other *Enterobacteriaceae* ^§^	7	5.6
	other non-fermenting Gram-negative bacteria ^#^	10	8.0
	*Vibrionaceae*	2	1.6
**Fungi**	*Candida* spp.	6	4.8
	*Aspergillus niger*	5	4

### 2.2. Susceptibility to Antibiotics

Table 2 shows the patterns of susceptibility to antibiotics of main pathogens. All *Enterobacteriaceae* exhibited resistance to ampicillin and to amoxicillin/clavulanate, and 14.3% showed resistance to ciprofloxacin and to trimethoprim/sulfamethoxazole. All strains (100%) resulted sensitive to cefotaxime, ceftazidime, cefepime, imipenem, gentamicin, and amikacin.

Imipenem and ciprofloxacin were the most active antibiotics against *P. aeruginosa* (100% sensitive strains), followed by amikacin (97%), cephalosporins (ceftazidime and cefepime) (87%), and gentamicin (84%).

Three *S. aureus* strains were resistant to cefoxitin and were considered Methicillin-Resistant *Staphylococcus aureus* (MRSA). Gentamicin, trimethoprim/sulfamethoxazole, linezolid and rifampicin (100% of sensitive strains) followed by vancomycin and ciprofloxacin (66.6%) were the most active molecules against MRSA. Rates of resistance of 67% to erythromycin were observed.

Other 11 *S. aureus* strains were susceptible to cefoxitin, and were considered Methicillin-susceptible *Staphylococcus aureus* (MSSA). All MSSA were susceptible to gentamicin, ciprofloxacin, trimethoprim/sulfamethoxazole, erythromycin, linezolid, and rifampicin (100% of susceptible strains). For vancomycin, rates of resistance of 27.3% were observed.

Coagulase-negative Staphylococci (CoNS) were sensitive to all tested antibiotics, with slight resistance to cefoxitin (46%).

**Table 2:** Percent susceptibility of bacterial strains to the major classes of antibiotics; MRSA = Methicillin-resistant *Staphylococcus aureus*; MSSA = Methicillin-susceptible *Staphylococcus aureus*; CoNS = coagulase-negative Staphylococci; * = Kirby-Bauer method; ** = *E*-test method; nt = not tested; AMP = ampicillin; AMC = amoxicillin/clavulanate; CTX = cefotaxime; CAZ = ceftazidime; FEP = cefepime; IMI = imipenem; GEN = gentamicin; AMK = amikacin; CIP = ciprofloxacin; SXT = trimethoprim/sulfamethoxazole; FOX = cefoxitin; ERI = erythromycin; VAN = vancomycin; LZN = linezolid; RIF = rifampicin.

Strains (*n*°)	AMP * (10 μg)	AMC * (20/10 μg)	CTX * (5 μg)	CAZ * (10 μg)	FEP * (30 μg)	IMI * (10 μg)	GEN * (10 μg)	AMK * (30 μg)	CIP * (5 μg)	SXT * (1.25/23.75 μg)	FOX * (30 μg)	ERI * (15 μg)	VAN **	LZD * (10 μg)	RIF * (5 μg)
*Enterobacteriaceae* (21)	0	0	90.5	90.5	95.2	100	100	100	85.7	85.7	nt	nt	nt	nt	nt
*P. aeruginosa* (31)	nt	nt	nt	87.0	87.0	100	84.0	97.0	100	nt	nt	nt	nt	nt	nt
MRSA (3)	nt	nt	nt	nt	nt	nt	100	nt	66.6	100	0	33.4	66.6	100	100
MSSA (11)	nt	nt	nt	nt	nt	nt	100	nt	100	100	100	100	72.7	100	100
CoNS (22)	nt	nt	nt	nt	nt	nt	86.0	nt	77.0	86.0	54.0	77.0	90.9	91.0	91.0

### 2.3. Biofilm Assay

Nine out of fourteen isolates of *S. aureus* and nine out of twenty-two CoNS were biofilm producers (Table 3). Seven out of thirty-one strains of *P. aeruginosa* were shown to be able to induce moderate biofilm production (slight producers), while only one strain was a biofilm producer.

**Table 3:** Production of biofilm by bacteria isolated from ear swabs. In brackets: number of strains. No. = number of strains and relative percentage (%). * = Congo Red Agar method; ** = Christensen method.

Strain	Producers		Slight Producers		Non Producers	
	No.	%	No.	%	No.	%
*S. aureus* (14) *	9	64	-	-	5	36
CoNS (22) (*)	9	41	-	-	13	59
*P. aeruginosa* (31) **	1	3	7	23	23	74
*E. coli* (8) **	2	25	3	37.5	3	37.5
*Klebsiella* spp. (7) **	0	0	6	86	1	14

*E. coli* strains were similarly distributed among producers, slight producers, and non-producers, while most of *Klebsiella* spp. were slight biofilm producers.

### 2.4. Antibacterial Activity of D. Tertiolecta and P. Subcapitata Extracts

Table 4 shows the MIC values obtained after treatment of main etiological agents of EO (*S. aureus*, *P. aeruginosa*, *E. coli*, *Klebsiella* spp.). MIC values obtained after treatment of *P. aeruginosa* and *S. aureus* strains with *D. tertiolecta* extract were included between 1.4 × 10^9^ and 5.6 × 10^9^ cells/mL and between 2.8 × 10^9^ and 1.1 × 10^10^ cells/mL, respectively. MIC values for *P. aeruginosa* and *S. aureus* strains exposed to *P. subcapitata* extract were included between 6.2 × 10^9^ and 1.2 × 10^10^ cells/mL and between 1.6 × 10^9^ and 1.2 × 10^10^ cells/mL, respectively.

**Table 4:** Antimicrobial activity of extracts from algae on selected pathogens. In brackets: number of strains. * Eight strains of *E. coli*; six strains of *Klebsiella* spp.; SD = Standard Deviation.

Algae	Pathogens	MIC cells\mL		
		MIC Range	MIC 50 (Mean ± SD)	MIC 90 (Mean ± SD)
*D. tertiolecta*	*S. aureus* (14)	2.8 × 10^9^–1.1 × 10^10^	5.6 × 10^9^ (5.6 × 10^9^ ± 0)	1.1 × 10^10^ (1.1 × 10^10^ ± 0)
	*P. aeruginosa* (31)	1.4 × 10^9^–5.6 × 10^9^	2.8 × 10^9^ (4.2 × 10^9^ ± 2.0 × 10^9^)	5.6 × 10^9^ (8.4 × 10^9^ ± 4.0 × 10^9^)
	*Enterobacteriaceae* (14) *	1.1 × 10^10^–2.2 × 10^10^	2.2 × 10^10^ (1.7 × 10^9^ ± 8.0 × 10^9^)	>2.2 × 10^10^ (2.3 × 10^10^ ± 0)
*P. subcapitata*	*S. aureus* (14)	1.6 × 10^9^–1.2 × 10^10^	6.2 × 10^9^ (4.7 × 10^9^ ± 2.2 × 10^9^)	1.2 × 10^10^ (7.8 × 10^9^ ± 6.6 × 10^9^)
	*P. aeruginosa* (31)	6.2 × 10^9^–1.2 × 10^10^	6.2 × 10^9^ (3.9 × 10^9^ ± 3.3 × 10^9^)	6.2 × 10^9^ (4.7 × 10^9^ ± 2.2 × 10^9^)
	*Enterobacteriaceae* (14)*	6.2 × 10^9^–1.2 × 10^10^	6.2 × 10^9^ (5.9 × 10^9^ ± 4.6 × 10^9^)	1.2 × 10^10^ (9.4 × 10^9^ ± 4.4 × 10^9^)

MIC 50 and MIC 90 values obtained after treatment of bacteria with *D. tertiolecta* extract were 5.6 × 10^9^ and 1.1 × 10^10^ cells/mL for *S. aureus*, 2.8 × 10^9^ and 5.6 × 10^9^ cells/mL for *P. aeruginosa*, 2.2 × 10^10^ cells/mL and >2.2 × 10^10^ cells/mL for enterobacteria, respectively. *D. tertiolecta* seemed to be a more potential resource to extract antimicrobial compounds against both major pathogens (*S. aureus* and *P. aeruginosa*), while *P. subcapitata* extract was more effective (MIC 50 = 6.2 × 10^9^ cells/mL; MIC 90 = 1.2 × 10^10^ cells/mL) against enterobacteria than *D. tertiolecta* extract.

## 3. Discussion

In this study, *S. aureus* and *P. aeruginosa* were the main agents of EO [5,27]. EAC mycoses had lower incidence (approx. 10%).

*P. aeruginosa* was the main pathogen in EO, notably during summer months. It can be supposed that the infection could be facilitated by hot and damp climate as well as by stagnation of water into the EAC due to bathing.

Through over one year sample collection from patients, what is known about the principal etiological agents of EO was confirmed, and a satisfactory susceptibility of isolated strains to antibiotics was shown. Therefore, notwithstanding the anatomical site is not favorable to drug diffusion, these results show that in normal conditions antibiotics can eradicate the pathogens.

To date, bacterial infections in the clinical practice are relevant to the biofilm formation by bacteria, and more than 60% of infections seem to be occurred with the presence of biofilms [28]. Recent studies showed that ear, nose, and throat (ENT) diseases are biofilm related [29]. In our study, 64% of *S. aureus* and 41% of CoNS isolated from ear swabs were biofilm producers. Among Gram-negative bacteria, 25.8% of *P. aeruginosa* were seen to be producers or slight producers of biofilm. Regarding *Enterobacteriaceae*, 77% of strains were producers or slight producers.

In this study, the evaluation of the antimicrobial activity of extracts from *D. tertiolecta* and *P. subcapitata* against the etiological agents of EO has been attempted. Results show that extracts from *D. tertiolecta* and *P. subcapitata* have antimicrobial activity, notably against *P. aeruginosa* (MIC 90 = 5.6 × 10^9^ and 6.2 × 10^9^cells/mL, respectively); lower activity was observed against *S. aureus* (MIC 90 = 1.1 × 10^10^ and 1.2 × 10^10^ cells/mL, respectively). The extracts, notably those from *P. subcapitata*, showed activity on *Enterobacteriaceae* as well.

*D. tertiolecta* extracts are known to contain polyphenols, notably gentisic acid, (+) catechin and (−) epicatechin [30]. Considering that the antimicrobial activity of polyphenols is already known [31], the activity of the extract can be supposed to be due to these compounds.

The interest of scientists for antimicrobials from microalgae started in early 1940s with the pioneering work of Pratt [32] who studied the antibacterial activity of chlorellin produced by *Chlorella*. Subsequently, Duff *et al.* [33] found remarkable antimicrobial properties in some Bacillariophyceae, Chrysophyceae and Cryptophyceae. Lustigman [34] stated that *Dunaliella* produces a broad spectrum of antibiotic substances depending on the site of collection. Notably, the antibiotic activity was observed in cultures isolated from high-polluted waters, presumptively as a result of a mechanism of survival in a highly competitive environment [34].

The crude extract from *Dunaliella primolecta* was shown to inhibit the growth of *S. aureus*, and a number of antibiotic substances was found in these algae [24]; α-linolenic acid, identified by NMR and GC-MS in extracts of *D. primolecta*, showed similar activity against MRSA [25].

Herrero *et al.* [21] and Mendiola *et al.* [22] evaluated the activity of carbon dioxide extracts of *Dunaliella salina* against *E. coli*, *S. aureus*, *C. albicans*, and *A. niger* detecting an indolic derivative and several volatile compounds and fatty acids provided with antimicrobial activity.

Furthermore, studying a panel of extracts of different microalgae on a panel of bacteria, Srinivasakumar and Rajashekhar [23] observed that the extract of *D. salina* did not affect the growth of *E. coli* and *S. aureus*, but the butanol extract was active on *P. aeruginosa*, and the chloroform extract was active on *K. pneumoniae*.

Recently, extracts of *D. tertiolecta* did not show activity against *E. coli*, *P. aeruginosa*, and *S. aureus*, but were effective against *Bacillus subtilis* [26].

Our results show that extracts from *D. tertiolecta* and *P. subcapitata* can yield compounds provided with antimicrobial activity useful for the treatment of otolaryngological diseases due to bacterial agents. Therefore, taking into account the present therapeutic difficulties due to the occurrence of resistant strains, the identification of the active molecule(s) can be a therapeutic perspective of high interest. Further studies will be needed to recognize the pharmacophores responsible for the antimicrobial activity and to elucidate the mechanisms of action of these algae extracts.

As a matter of fact, a number of compounds having interesting pharmacological characteristics have been discovered in marine organisms [35] and the research in this field is in progress. Notably, marine and freshwater microalgae are known to be a rich source of chemically diverse compounds having a potential use as bioactive compounds and antimicrobial agents [36,37,38,39]. In addition, biotechnological studies on transgenic microalgae evidenced the protective activity of newly produced peptides against infections of fish digestive tract [40], making this research useful not only in human pathology but also in aquaculture.

Thus, the biomass derived from microalgae is considered a source of valuable chemical constituents that can have application in human and animal nutrition, in agriculture, and in pharmaceutical and cosmetics industries. Therefore, the development of biotechnological tools to ensure the supply of algal biomass of sufficient quality and quantity is desirable to meet the growing demand [41].

In human pathology the recurrent antibiotic-resistance observed in the treatment of several infectious diseases is a challenge for the modern pharmacology. On the basis of what reported above and considering the antibiotic-resistance of bacterial strains, the increased occurrence of nosocomial infections, and the consequent increasing request of new antimicrobials for the treatment of infectious diseases, the research about antimicrobials originating from algae may be in the coming future a valuable therapeutic support opening new perspectives for the utilization of new and still unexploited sources of drugs.

## 4. Experimental Section

### 4.1. Pathogenic Micro-Organisms Collection

For 13 months (from January 2012 to January 2013), 100 ear swabs were collected from patients with suspected EO, seen at the First Aid Department of IRCSS Azienda Ospedaliera Universitaria San Martino-IST, Genova. Ear swabs were sent to the Microbiology Laboratory of DISC (University of Genova) for further analysis. All consecutive, non-duplicates strains were studied.

### 4.2. Bacterial Strains and Growth Conditions

Ear swabs were plated in Columbia horse blood agar, and in appropriate selective culture medium (Mc Conkey agar for Gram-negative bacteria, Mannitol Salt agar for Staphylococci, Sabouraud agar for fungi) (Biolife, Milano, Italy). The plates were incubated at 37 °C for 18–24 h. The incubation of Sabouraud plates was prolonged up to a week.

All isolates were identified to the species level by routine methods, and an API STAPH, API20E, API NE and API STREP system (bioMèrieux, Marcy l’Etoile, France) for Staphylococci, *Enterobacteriaceae*, non-*Enterobacteriaceae*, and Streptococci respectively.

The antibiotype (excepting vancomycin) was determined using the disk diffusion test, according to the latest Clinical and Laboratory Standards Institute (CLSI) guidelines [42,43] and was interpreted according to the European Committee for Antimicrobial Susceptibility Testing [44]. The MICs of Vancomycin were determined by *E*-test (bioMérieux, Marcy-l’Etoile, France) on Muller-Hinton agar (Biolife, Milano, Italy). Cefoxitin disk diffusion test was used to confirm oxacillin resistance [44].

*S. aureus* ATCC-29213, *E. coli* ATCC-25922, and *P. aeruginosa* ATCC-27853 were included for the quality control of antimicrobial susceptibility patterns.

### 4.3. Biofilm Assay

The searching of biofilm producers was carried out on strains of *S. aureus*, *P. aeruginosa*, *E. coli* and *K. pneumoniae*.

The presence and extent of biofilms produced by bacteria was quantified spectrophotometrically at 570 nm using the microtitre-plate test (MtP), according to Christensen *et al.* [45] and Roveta *et al.* [46]. Strains with optical density (OD) values ≤0.120 were considered negative, and those with ODs >0.120 and <0.240 were regarded as slight biofilm-producers. An OD value ≥0.240 was indicative of biofilm production. For each isolate the MtP test was repeated in triplicate.

The phenotypic characterization of slime-producing ability of Staphylococci was also carried out by culturing strains on Congo Red Agar (CRA) plates, prepared by adding 0.8 g of Congo Red and 36 g of saccharose (Sigma, St. Louis, MO, USA) to 1 L of Brain Heart infusion agar (Oxoid, Basingstoke, Hampshire, UK) as described by Freeman *et al.* [47]. *S. aureus* colonies on CRA were kept under observation for up to 72 h, as described by Arciola *et al.* [48].

### 4.4. Marine and Freshwater Algae

Marine algae *Dunaliella tertiolecta* (*Chlorophyta; Chlorophyceae*) were cultured in filtered sterile seawater (Millipore 0.2 μm) supplemented with Walne medium and B1–B12 vitamin solution [49]. Algae were grown in a refrigerated incubator (BK6160 Heraeus Instruments GmbH, Hanau, Germany), at 20 ± 0.5 °C and 16:8 h light:dark (L:D). Freshwater algae *Pseudokirchneriella subcapitata*, formerly *Selenastrum capricornutum* (*Chlorophyta: Chlorophyceae*), were grown in proper culture medium [18] in a refrigerated incubator (BK6160 Heraeus Instruments GmbH, Hanau, Germany), at 22 ± 0.5 °C and 16:8 h L:D. Both cultures were prepared from axenic stocks and growth was evaluated by cell counting (Thoma hemocytometer, Brand GmbH, Wertheim, Germany).

Samples of *D. tertiolecta* and *P. subcapitata* were counted and concentrated by centrifugation at 3500 rpm for 30 min. Subsequently, the pellet was washed with PBS (*D. tertiolecta*) or phosphate buffer (*P. pseudocapitata*). The resulting samples were sonicated (twenty cycles of 30 s each) with a Soniprep (Cellai, Milano, Italy) sonicator, and re-centrifuged at 3500 rpm for 30 min. A 60% methanol was added to the pellet in a ratio of the initial algae sample of 5 g to 20 mL of methanol. The resulting sample was re-centrifuged at 3500 rpm for 20 min. The supernatant was then filtered with 0.22 μm filters (Millipore GV, Billerica, MA, USA) and the resulting methanol extract was utilized. The results of antimicrobial activity are expressed as algal cells/mL, considering the number of algal cells counted at the outset of the procedure.

### 4.5. Bacterial Susceptibility (MIC Determination)

Minimum Inhibitory Concentration (MIC) of extracts from *D. tertiolecta* and *P. subcapitata* was determined using the broth microdilution method, according to the CLSI guidelines [42,43]. Briefly, algae extracts have been tested on cultures of bacteria (5 × 10^5^ CFU per mL) in 96-well microtiter plates in Mueller-Hinton broth. After 18–24 h of incubation at 37 °C, the concentration of the extract from marine algae which prevented a visible bacterial growth was identified as the MIC. All tests were performed in triplicate and were executed three times.

## References

[1] Rosenfeld R.M., Brown L., Cannon C.R., Dolor R.J., Ganiats T.G., Hannley M., Kokemueller P., Marcy S.M., Roland P.S., Shiffman R.N. (2006). Clinical practice guideline: Acute otitis externa. Otolaryngol. Head Neck Surg..

[2] Sander R. (2001). Otitis externa: A practical guide to treatment and prevention. Am. Fam. Phys..

[3] McWilliams C.J., Smith C.H., Goldman R.D. (2012). Acute otitis externa in children. Can. Fam. Phys..

[4] Pabla L., Jindal M., Latif K. (2012). The management of otitis externa in UK general practice. Eur. Arch. Otorhinolaryngol..

[5] Jayakar R., Sanders J., Jones E. (2014). A study of acute otitis externa at Wellington Hospital, 2007–2011. Australas. Med. J..

[6] Bhattacharyya N., Kepnes L.J. (2011). Initial impact of the acute otitis externa clinical practice guideline on clinical care. Otolaryngol. Head Neck Surg..

[7] Albera R., Rossi G. (2012). Otorinolaringoiatria.

[8] Mösges R., Nematian-Samani M., Eichel A. (2011). Treatment of acute otitis externa with ciprofloxacin otic 0.2% antibiotic ear solution. Ther. Clin. Risk Manag..

[9] Schaefer P., Baugh R.F. (2012). Acute otitis externa: An update. Am. Fam. Phys..

[10] Newman D.J., Cragg G.M., Snader K.M. (2000). The influence of natural products upon drug discovery. Nat. Prod. Rep..

[11] Proksch P., Edrada-Ebel R.A., Ebel R. (2003). Drugs from the sea—Opportunities and obstacles. Mar. Drugs.

[12] Newman D.J., Cragg G.M., Snader K.M. (2003). Natural products as sources of new drugs over the period 1981–2002. J. Nat. Prod..

[13] Faulkner D.J. (1994). Marine natural products. Nat. Prod. Rep..

[14] Fenical W., Jensen P.R., Attaway D.H., Zaborsky O.R. (1993). Marine microorganisms: A new biomedical resource. Marine Biotechnology. Pharmaceutical and Bioactive Natural Products.

[15] Shmizu Y. (1993). Microalgal metabolites. Chem. Rev..

[16] Shimizu Y., Attaway D.H., Zaborsky O.R. (1993). Dinoflagellates as sources of bioactive molecules. Marine Biotechnology. Pharmaceutical and Bioactive Natural Products.

[17] Butcher R.W. (1959). Part I: Introduction and Chlorophyceae, Ser. IV. An Introductory Account of the Smaller Algae of British Coastal Waters.

[18] Passarelli P., Sbalchiero A. (2005). Test di Inibizione Algale con *Selenastrum capricornutum* o *Pseudokirchneriella subcapitata*. Notiziario dei Metodi Analitici N.1.

[19] Orias F., Simon L., Perrodin Y. (2015). Experimental assessment of the bioconcentration of 15*N*-tamoxifen in *Pseudokirchneriella subcapitata*. Chemosphere.

[20] Pane L., Mariottini G.L., Giacco E. (2015). Ecotoxicological assessment of the micelle encapsulator F-500. Ecotoxicol. Environ. Saf..

[21] Herrero M., Ibáñez E., Cifuentes A., Reglero G., Santoyo S. (2006). *Dunaliella salina* microalga pressurized liquid extracts as potential antimicrobials. J. Food Prot..

[22] Mendiola J.A., Santoyo S., Cifuentes A., Reglero G., Ibáñez E., Señoráns F.J. (2008). Antimicrobial activity of sub- and supercritical CO_2_ extracts of the green alga *Dunaliella salina*. J. Food Prot..

[23] Srinivasakumar K.P., Rajashekhar M. (2009). *In vitro* studies on bactericidal activity and sensitivity pattern of isolated marine microalgae against selective human bacterial pathogens. Indian J. Sci. Technol..

[24] Chang T., Ohta S., Ikegami N., Miyata H., Kashimoto T., Kondo M. (1993). Antibiotic substances produced by a marine green alga, *Dunaliella primolecta*. Bioresour. Technol..

[25] Ohta S., Shiomi Y., Kawashima A., Aozasa O., Nakao T., Nagate T., Kitamura K., Miyata H. (1995). Antibiotic effect of linolenic acid from *Chlorococcum* strain HS-101 and *Dunaliella primolecta* on methicillin-resistant *Staphylococcus aureus*. J. Appl. Phycol..

[26] Sánchez-Saavedra M.P., Licea-Navarro A., Bernáldez-Sarabia J. (2010). Evaluación de la actividad antibacteriana de diferentes especies de fitoplancton. Rev. Biol. Mar. Oceanogr..

[27] Kaushik V., Malik T., Saeed S.R. (2010). Interventions for acute otitis externa. Cochrane Database Syst. Rev..

[28] Stewart P.S., Costerton J.W. (2001). Antibiotic resistance of bacteria in biofilms. Lancet.

[29] Vlastarakos P.V., Nikolopoulos T.P., Maragoudakis P., Tzagaroulakis A., Ferekidis E. (2007). Biofilms in ear, nose, and throat infections: How important are they?. Laryngoscope.

[30] Lòpez A., Rico M., Santana-Casiano J.M., Gonzàlez-Dàvila M. (2015). Phenolic profile of *Dunaliella tertiolecta* growing under high levels of copper and iron. Environ. Sci. Pullut. Res. Int..

[31] Coppo E., Marchese A. (2014). Antibacterial activity of polyphenols. Curr. Pharm. Biotechnol..

[32] Pratt R.H. (1942). Studies on *Chlorella vulgaris* V: Some properties of the growth inhibitor formed by Chlorella cells. Am. J. Bot..

[33] Duff D.C.B., Bruce D.L., Antia N.J. (1966). The antibacterial activity of planktonic algae. Can. J. Microbiol..

[34] Lustigman B. (1988). Comparison of antibiotic production from four ecotypes of the marine alga, *Dunaliella*. Bull. Environ. Contam. Toxicol..

[35] Mayer A.M.S., Rodríguez A.D., Taglialatela-Scafati O., Fusetani N. (2013). Marine pharmacology in 2009–2011: Marine compounds with antibacterial, antidiabetic, antifungal, anti-inflammatory, antiprotozoal, antituberculosis, and antiviral activities; affecting the immune and nervous systems, and other miscellaneous mechanisms of action. Mar. Drugs.

[36] Katircioglu H., Beyatli Y., Aslim B., Yüksekdag Z., Atici T. (2005). Screening for antimicrobial agent production of some microalgae in freshwater. Internet J. Microbiol..

[37] Amaro H.M., Guedes A.C., Malcata F.X., Méndez-Vilas A. (2011). Antimicrobial activities of microalgae: An invited review. Science Against Microbial Pathogens: Communicating Current Research and Technological Advances.

[38] Sanmukh S., Bruno B., Ramakrishnan U., Khairnar K., Swaminathan S., Paunikar W. (2014). Bioactive compounds derived from microalgae showing antimicrobial activities. J. Aquac. Res. Dev..

[39] Encarnação T., Pais A.A.C.C., Campos M.G., Burrows H.D. (2015). Cyanobacteria and microalgae: A renewable source of bioactive compounds and other chemicals. Sci. Prog..

[40] Li S.S., Tsai H.J. (2009). Transgenic microalgae as a non-antibiotic bactericide producer to defend against bacterial pathogen infection in the fish digestive tract. Fish Shellfish Immunol..

[41] Stengel D.B., Connan S. (2015). Marine algae: A source of biomass for biotechnological applications. Method Mol. Biol..

[42] Clinical and Laboratory Standards Institute (CLSI) (2012). Performance Standards for Antimicrobial Disk Susceptibility Tests; Approved Standard—Eleventh Edition. Document M02-A11.

[43] Clinical and Laboratory Standards Institute (CLSI) (2012). Methods for Dilution Antimicrobial Susceptibility Tests for Bacteria That Grow Aerobically; Approved Standard—Ninth Edition. Document M07-A9.

[44] European Committee on Antimicrobial Susceptibility Testing (EUCAST) (2014). Breakpoint Tables for Interpretation of MICs and Zone Diameters. http//www.eucast.org/clinical_breakpoints/.

[45] Christensen G.D., Simpson W.A., Younger J.J., Baddour L.M., Barrett F.F., Melton D.M., Beachey E.H. (1985). Adherence of coagulase-negative *staphylococci* (CoNS) to plastic tissue culture plates: A quantitative model for the adherence of *staphylococci* to medical devices. J. Clin. Microbiol..

[46] Roveta S., Marchese A., Schito G.C. (2008). Activity of daptomycin on biofilms produced on a plastic support by *Staphylococcus* spp.. Int. J. Antimicrob. Agents.

[47] Freeman D.J., Falkiner F.R., Keane C.T. (1989). New method for detecting slime production by coagulase negative *staphylococci*. J. Clin. Pathol..

[48] Arciola C.R., Baldassarri L., Montanaro L. (2001). Presence of icaA and icaD genes and slime production in a collection of staphylococcal strains from catheter-associated infections. J. Clin. Microbiol..

[49] Walne P.R. (1966). Experiments in the large-scale culture of the larvae of *Ostrea edulis* L.. Fishery Investigations Series II.

